# The global regulatory framework for the commercialization of nutrient enriched biofortified foods

**DOI:** 10.1111/nyas.14869

**Published:** 2022-08-29

**Authors:** Tora Mitra‐Ganguli, Wolfgang H. Pfeiffer, Jenny Walton

**Affiliations:** ^1^ Path2Health Consulting Ltd. West Kirby United Kingdom; ^2^ HarvestPlus Organization Washington DC USA

**Keywords:** biofortification, biofortified, conventional breeding, food regulations, food standards, HarvestPlus, healthy food marketing, nutrient enriched crops, publicly available standards

## Abstract

Nutrient enriched crops (NECs) were developed through biofortification as a tool to reach the world's most vulnerable. The delivery model developed by HarvestPlus for the scaling of NECs relies on commercial demand from food businesses and consumers, coupled with the ability to promote and market foods that comply with legislation. This review of standards, regulations, and laws across the value chain in 20 countries demonstrates that existing provisions for food labeling are sufficient to carry out sales and marketing of foods made from conventionally bred NECs. The term biofortification is not necessary to create demand and, potentially, is counterproductive. Promoting the natural source of vitamins and minerals and relevant nutrition claims is the most effective and simple way to signpost healthier products to consumers. Until 2021, it was not possible to distinguish NECs at the grain level from the market standard. The development of a globally relevant Publicly Available Specification allows traders to demand grains that offer a substantial increase in zinc, iron, or vitamin A. Addressing this gap at the grain level ensures that standards and regulations are available end‐to‐end in the food supply chain providing the enabling environment for the rapid scale of NECs.

## Graphical abstract

Nutrient enriched crops (NECs) developed through biofortification were developed as a tool to reach the world's most vulnerable. The delivery model developed by HarvestPlus for the scaling of NECs relies on commercial demand from food businesses and consumers coupled with the ability to promote and market foods that comply with legislation. This review of standards, regulations, and laws across the value chain in 20 countries demonstrates that existing provisions for food labeling are sufficient to carry out sales and marketing of foods made from conventionally bred NECs.

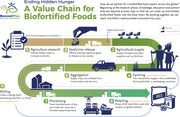

## INTRODUCTION

### Overview of biofortification

Biofortification is defined by WHO as:

the process by which the nutritional quality of food crops is improved through agronomic practices, conventional plant breeding, or modern biotechnology. Biofortification differs from conventional fortification in that biofortification aims to increase nutrient levels in crops during plant growth rather than through manual means during processing of the crops.^1^

In broad terms, biofortification can cover anything from increasing nutrients in animal feeds that transfers to eggs, milk, and meat, genetic engineering of rice to introduce a gene for vitamin A,^2^ or increasing the vitamin D content of mushrooms through UV‐light or sunlight exposure.^3^ Biofortification is a process that can cover any category of food at many points in the food supply chain before harvest to increase the naturally occurring nutrient content of foods so that further addition of nutrients postharvest is not necessary. HarvestPlus is part of the global Consultative Group for International Agricultural Research (CGIAR), a not for profit, donor‐funded organization with the mission to improve nutrition, health, and livelihoods to develop and promote biofortified crops that are rich in vitamins and minerals and are climate smart. HarvestPlus provides leadership on biofortification evidence and technology. Although biofortification of crops through breeding has been used for centuries, the formal term *biofortification* was introduced by the HarvestPlus project after 2003. The ambition of the HarvestPlus project is to reach 1 billion consumers worldwide by 2023; this can only be achieved if biofortified staple foods are embedded in commercial food systems and supply chains. Standards and regulations distinguish biofortified foods from the regular variety and, therefore, are essential in the scale and commercial utilization of biofortified produce.

The success of biofortification hinges on the commitment of leading public and private research to increase the amounts of iron, zinc, and vitamin A in most common foods. Biofortification is the basis of four complementary interventions—including dietary diversity, large‐scale food fortification, and supplementation—that in tandem have the potential to eradicate hidden hunger on a global scale.

The HarvestPlus program aims to improve the content of the nutrients of global concern in the foods that people rely on. It is staggering to note that while the planet has ∼300,000 edible plant species, humans consume only ∼200;^4^ and 60% of the calories consumed by humans are from wheat, maize, and rice.^5^ This lack of dietary diversity is a major cause that one quarter of the world population suffers from hidden hunger (micronutrient malnutrition). In the past, plant breeding in both the public and private sectors focused on yield and pest resistance. The green revolution of the 1950s and 1960s concentrated on calories in avoiding a Malthusian catastrophe; little attention was paid to micronutrients, which by then were not recognized as value‐added traits. Until very recently, breeders unintentionally compromised micronutrients by developing crops with lower protein/starch ratios. The HarvestPlus program began with the intention to reach the world's most vulnerable consumers, who generally grow their own crops for household consumption; but it was soon realized that to scale biofortified seeds, grains, and foods, wider demand would be required in the full food system. Gaining access to urban markets and higher‐value processed food markets is critical for sustained and accelerated adoption of biofortified products, even if some of the consumers targeted do not have the greatest nutritional need. Diversified use and income potential are important along with improving nutritional status. Some of the crops, for example, beans and vegetative crops, begin with a noncommercial planting material system, and, therefore, standards are not deemed essential in these noncommercial seed systems; however, once the crop is ready, it then becomes a commercial commodity, which relies on standards for trade.

**FIGURE 1:**
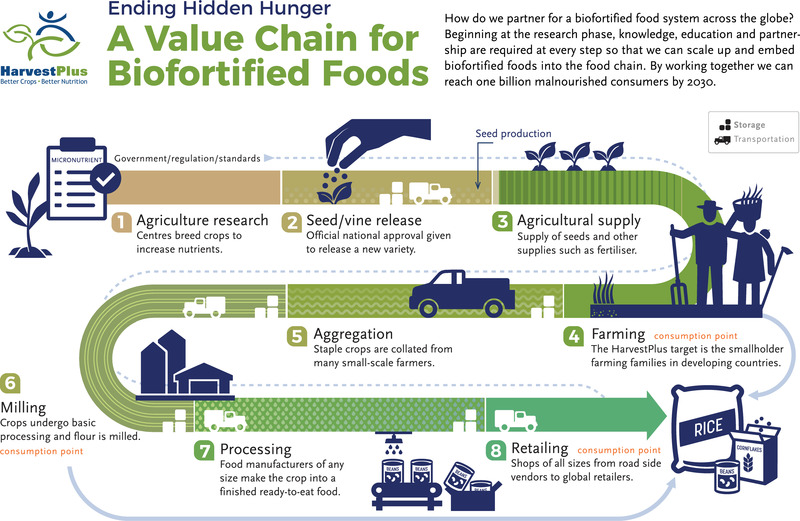
Value chain for biofortified foods.

Biofortification spans the entire value chain, from agricultural research to consumption points on farm, to the market shopper (Figure 1). To embed biofortified seeds, grains, and foods into food systems standards, regulations and laws are critical to enable trade at each value chain point. The ultimate priority is to protect the on‐ and off‐farm consumer and deliver foods to beneficiaries that make a genuine and measurable impact on health.

**FIGURE 2:**
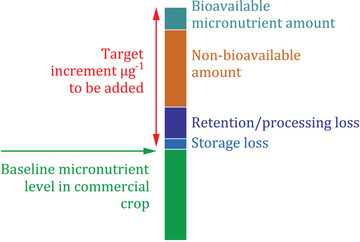
Establishing target increments.^68^, ^69^

A previous review of the global regulatory frameworks for biofortified crops^6^ concluded that “the production and marketing of these [biofortified] products have been conducted without regulatory framework and under limited government control or regulatory guidance” and that “regulatory considerations are rare or nonexistent.” However, our review here finds that biofortification, through conventional or genetic modification (GM), has adequate regulatory frameworks to label and promote foods made from nutrient enriched crops (NECs). Previous efforts to define biofortification were leading researchers in the wrong direction. Mejia et al.^6^ also concluded that

as all biofortified products derived from conventional breeding are no more than selected crop varieties naturally rich in certain micronutrients, the use of specific standards and regulations may be unnecessary beyond compliance with food quality and general safety requirements on existing food legislation, including proper labeling. However, when the current scenario expands and becomes diversified including other biofortification techniques, such as agronomic fertilization and GM, including the NBTs, an appropriate regulatory framework for biofortified foods will become necessary, covering all stages of implementation.

The generic term for regulatory framework is used in this paper, but what was found is a combination of private standards, public standards, public or government policies, regulations, and laws exist simultaneously and are collectively referred to as *regulatory frameworks*.

### Global and local definitions of biofortification and nutrient enriched crops

The term *biofortification* was introduced by Steve Beebe, a bean researcher at the International Centre for Tropical Agriculture (CIAT), at a meeting convened in early 2001 by CIAT. The meeting aimed to inform representatives of the Bill and Melinda Gates Foundation (BMGF) and the Micronutrient Initiative (now Nutrition International) about the CGIAR Micronutrients Project and the “biofortification” strategy.^7^ The terminology caught on in the field of research and has been used ever since. As the HarvestPlus program escalated, more crops developed and more governments embraced the practice, the WHO then developed working terminology.^8^ There are several definitions of “biofortification” for different purposes. The following definition is from USDA's National Agricultural Library Agricultural Thesaurus

Biofortification, an increase in the nutritional value of plant foods obtained through conventional crop breeding methods or through crop genetic engineering techniques. This contrasts with postharvest fortification in which nutrients are added during processing.^9^

Donors and practitioners in the HarvestPlus program believed that the lack of definition would pose a barrier to scale. The Codex Alimentarius commission was engaged to discuss the definition and led by Zimbabwe and South Africa, an Electronic Working Group (EWG) was formed.^10^, ^11^

Following several years of EWG discussion and member consultation at the CCNFSU41 meeting on November 26, 2019, the committee agreed to discontinue the work. The concluding remarks of the meeting refer to Nutrition and Health claims (CAC/GL 23–1997) and that the nutrient reference values (NRVs) for nutrition labeling should be used for biofortified foods where appropriate. During the process, member states had raised concerns about the suffix “bio,” meaning organic in several countries and possibly creating confusion with genetic engineering standards and regulations that were already in place. In the years that were spent in the codex negotiations, the delivery of seeds, grains, and foods kept growing;^12^ thus, the scaling of biofortified foods was not held back by lack of a definition.

### Stakeholder research in 2018

Stakeholder research has revealed other barriers to scale.^13^ Following interviews with almost 100 seed, grain, and food businesses as well as 250 stakeholders, it was clear that supply chain actors were unaware of standards, regulations, or laws that governed trade and marketing of biofortified commodities. While academic publications set breeding targets for the levels of nutrients required,^14^ this was of academic nature, and standards for trade were not available. Several publications at the time, including Mejia *et al*.,^6^ concluded that “the production and marketing of these [biofortified] products have been conducted without regulatory framework and under limited government control or regulatory guidance.” This assumption was based on the lack of definition, and at the time of publication, the codex affirmation stated that existing food labeling provisions, Codex texts, standards, and regulations already covered biofortified grains and foods and products thereof.

Not so much the lack of an agreed definition of the term biofortification but the actual use of the word caused issues for stakeholders. Opinions from leading global regulatory experts^15^ concluded that given that the term biofortification had no regulatory status and was not a regular term used by consumers, it would be misleading and confusing for food product labeling and, therefore, should be avoided in packaging and promotion.^16^

The word biofortification is still used in academic and policy documents, but outside of these subject areas, a better description was required. The Food Fortification Advisory Services (2FAS) is an advisory service funded by the European Union (EU) and implemented through a partnership between Landell Mills and the Global Alliance for Improved Nutrition.^17^ In 2019, 2FAS developed a new term *nutrient enriched crop*, which are crops grown to have an enhanced nutritional value. Unlike other forms of fortification, in which vitamins and minerals are added to foods manually and postharvest, NECs are cultivated to have a higher nutrient content. This is achieved through agricultural practices or plant breeding.^18^

GM (or genetic engineering or biotechnology) is a tightly regulated area of food production covered by detailed Codex guidelines,^19^ European Union Regulations,^20^ and the USFDA.^21^ We shall discuss only NECs developed through conventional breeding technologies. NECs derived from any other technologies are subject to different and specific food labeling codes and international laws.

It has been recommended not to use the term *biofortification* in any consumer facing materials^16^ and to describe NECs specifically by the nutrients they provide when they meet the specific recommended daily amounts (RDAs) for the nutrient according to Codex or national provisions. For example, biofortified beans with an elevated level of iron, which contain 15% of the NRV of iron per 100 g, should be called “iron beans.” When the term biofortification is removed from the discussion, a regulatory review takes on a new form. Foods made from conventionally bred biofortified crops/grains are no different to standard crops except for the increased amount of a specific micronutrient, notably, iron, zinc, or vitamin A. Therefore, the foods fall under the jurisdiction or codes for all other foods.

## METHODOLOGY OF REVIEW OF THE REGULATORY FRAMEWORKS FOR SEEDS, GRAINS, AND FOODS

The biofortification value chain is long (Figure 1), and regulatory provisions are required at each step of the value chain. Our review focuses on the available provisions for conventionally bred crops or those produced through agronomic biofortification. Further research is required for crops developed through genetic engineering, for example, such crops as golden rice. To date, all crops delivered through the HarvestPlus program have been developed through conventional breeding, and our review here was carried out to enable scale through commercialization of these products. HarvestPlus has active delivery programs in several countries; these are deemed priority markets for commercialization. Other developed countries and markets are discussed to consider the role of trade and market expansion for biofortification, including Africa (Democratic Republic of the Congo, Kenya, Nigeria, Tanzania, Uganda, Rwanda, Zambia, and Zimbabwe); Asia (Bangladesh, India, and Pakistan); Europe (United Kingdom and European Community); North America (the United States); and Latin America (Brazil, Colombia, Guatemala, El Salvador, Honduras, and Nicaragua).

For each of the countries listed above, the following questions were asked. (1) Is there a legal, academic, policy, or working definition of the term biofortification in specific country documents? (2) Does the country have policy support for biofortification, nutrition‐sensitive agriculture, or NECs in agriculture, public health, institutional feeding, or public health policy? (3) Does the country have specific seed standards or protocols or country advice to follow global schemes? (4) Does the country have specific standards or country advice to follow global standards for the nutrient content of grains? (5) Has the country established food standards for nutrition and health claims? (6) Do the regulations cover the following elements: RDAs for iron, zinc, and vitamin A? and provide provision of the claim “natural source” of iron, zinc, and vitamin A? (6) Are there existing policies, regulations, or standards governing fortification and or biofortification?

Information was gathered in partnership with Leatherhead Food Research (https://www.leatherheadfood.com/) and experts at HarvestPlus country offices, especially in Latin America. In many cases, local government officials were interviewed. Policies, regulations, and standards were uncovered and identified using search engines and websites of health and nutrition organizations and nongovernmental organizations and scientific databases, including PubMed and websites of health‐ and nutrition‐related organizations, such as the WHO and Codex Alimentarius. International regulatory information was searched on the Global database on the implementation of Nutrition Action (GINA) (https://extranet.who.int/nutrition/gina/en).

A comprehensive analysis of regulations covering fortification was also included, as biofortification is increasingly viewed as preharvest fortification. Governments and thought leaders in the areas of public health and nutrition increasingly view any micronutrient interventions through a more holistic complementary lens and, therefore, policies and position statements often include biofortification—prime examples being India and Nigeria and Zimbabwe. Table 1 shows the existing standards and regulations for fortification and biofortification available in various regions across the world.

**TABLE 1:** Overview of standards and regulations for fortification and biofortification

	Local definition of biofortification	Seed standard system	Grain standards	Food standards	NRV or DRV for labeling	Natural source of nutrient claim permitted	Fortification regulations
Africa							
°DRC	Yes^22^	Yes	PAS	Codex	Codex	Codex	In progress
°Kenya	Yes^23^	Yes	PAS	Yes^24^, ^25^	Yes^24^, ^25^	Yes^24^, ^25^	Yes^26^
°Nigeria	Yes^27^	Yes	PAS	Yes^28^	Yes^28^	Yes^28^	Covers biofortification^29^
°Tanzania	Yes^30^	Yes	PAS	Codex	Codex	Codex	Yes^31^
°Uganda	Yes^32^	Yes	PAS	Yes^33^	Yes^34^	Yes^35^	Yes^36^
°Rwanda	Yes^37^	Yes	PAS Rwanda standard	Yes^38^, ^39^	Yes^38^, ^39^	Yes^38^, ^39^	Yes^40^
°Zambia	Yes^41^	Yes	PAS	Yes^41^	Yes^42^	Yes^42^	Yes^43^, ^44^
°Zimbabwe	Yes^45^	Yes	PAS	Yes^45^	Yes^45^	Yes^46^	Covers biofortification published in 2022^47^
Asia							
°Bangladesh	Yes^48^	Yes	PAS	Yes^49^	Yes^50^	Yes^50^	Yes, for Vit A^51^
°India	Yes^52^	Yes	PAS	Yes^53^	Yes^54^	Yes^54^	Covers biofortification^55^
°Pakistan	Yes^56^	Yes	PAS	Yes^57^	Yes^58^	Yes^58^	Yes^58^
Europe							
°European Commission	Yes^59^	Yes	PAS	Yes^60^	Yes^61^	Yes^61^	Covers biofortification^62^
°United Kingdom	Yes^63^	Yes	PAS	Yes^64^	Yes^61^	Yes^61^	Yes^65^
Latin America							
°Columbia	Yes^66^	Yes	PAS	Yes^67^	Yes^67^	Yes^67^	Yes

Abbreviation: PAS, publicly available specification.

## RESULTS

### Government policy

Our paper is not a systematic review of country policy for support for biofortification, nutrition‐sensitive agriculture, or NECs in agriculture, public health, institutional feeding, or public health policy. Policy documents were found for established local use of definitions of biofortification. There is large‐scale policy support from governments from agricultural practice and guidelines to institutional feeding procurement standards. Increasingly, governments are viewing the range of available interventions to tackle micronutrient deficiencies with a more holistic and complementary approach. The best examples of countries having an integration of interventions are, as mentioned above, India,^52^, ^53^, ^54^, ^55^ Nigeria,^27^, ^28^, ^29^ and Zimbabwe.^45^, ^46^, ^47^ Once a government endorses the practice of biofortification, the seed, grain, and food industries are more likely to adopt it. The BMGF is a major sponsor of work on both expanding policy coverage and implementation guidelines. Scaling NECs is also achieved by catalyzing governments to integrate biofortification in agriculture and health policies, programs, regulations, and standards and varietal release protocols. As a result of the work to demonstrate impact evidence to governments (advocacy), biofortification has been included in 24 countries’ national health and agriculture strategies and policies, with many more to be published in 2022.

### Breeding standards

Crop breeding is the basic and most critical part of implementing biofortification programs. Biofortification was originally intended to be a public health tool, with basic but fundamental calculations on typical daily intake of a given staple, then the amount of nutrient required to achieve adequate micronutrient intake, “[b]iofortification improves status for those less deficient and maintains status for all at low cost. Biofortification will shift the population into a more sufficient range.”^14^ This localized consumption‐level approach is crop/country context specific. These fundamental principles of biofortification were developed in 2005 by a consultative interdisciplinary working group in 2005 and first published in 2010.^14^

It is critical to note that breeding targets and target increments are based on baseline levels of nutrients. As biofortification aims to increase levels of existing nutrients, standard maize, for example, has very low amounts of provitamin A, and so a target increment has to be added to the specific baseline to achieve a measurable impact to human health. To describe the baseline level, the following text from the Publicly Available Specification (PAS) for zinc has been used,

the average content of a micronutrient, such as iron and several other trace elements, might vary country to country and even within a large country due to different agro‐ecological zones. These natural variations occur and are based on factors external to the plant, including soil health, plant maturation, climate conditions, water availability, and several other environmental and biological elements. Select germplasms might also have naturally higher levels. Due to these variations, a global baseline value is established to set uniform standards to assist breeding programs, governments, and industries implement and scale biofortification.^68^

On a targeted basis, it is easier to use one given baseline for one crop in one country, but on a global scale with multiple crops and nutrients, it becomes increasingly different, and the baselines will change over time.^68^, ^69^ All standards require a benchmark, and these globally accepted global baselines (or averages) are used for breeding standards and, therefore, are transferred to seed and grain standards. It is essential that periodic reviews of baselines occur, and standards are upgraded accordingly. The use of the baseline and consideration of external factors is summarized below in the diagram (Figure 2) on how to establish target increments for given crops.

While these breeding standards have been published and widely circulated and adopted in agricultural research, they have not been viewed as standards which could be used outside of academia. The standards are the basis for government‐led breeding and seed targets and the seed, grain, and food targets thereafter. The most important premise is to breed plants that have the genetic potential to demonstrably change the nutritional intake and status of the target population. These principles must encompass many other biological issues, such as bioavailability and processing losses through the supply chain (including storage and shelf‐life issues).

As the science of biofortification expands and as more governments recognize the importance of biofortification as an effective nutrition‐sensitive agricultural intervention, it will become more frequent in individual country policy and among seed breeders in the public sector. Food businesses are increasingly recognizing the consumer demand for natural clean labels (minimally added ingredients) and are beginning to demand nutrient enriched grains from their suppliers. With the demand in the private sector and the public health needs in the public sector, nutritional targets for micronutrients will increasingly be part of the norm, from crop breeding targets to mainstream food systems for production of foods. Mainstreaming in the context of biofortification has been reviewed by experts in the CGIAR, and breeders will have an integrated push for mainstreaming nutrition through simultaneous selection for micronutrients as core traits of interest in future breeding.^70^

To ensure adoption by agricultural researchers in the public and private sector, micronutrient content should be a mandatory consideration and allocated equal importance as yield. Governments play a critical role at this first step of the value chain by including these breeding standards in agricultural policy, the best example found for this is India,^71^ where mandatory minimum threshold levels for pearl millet variety have been established. It remains unsettled whether these breeding standards should become legal requirements or adopted by the public and private sector. As nutritional breeding standards are adopted more freely, it will be easier to translate breeding targets into the food products and nutrition claims. If breeding standards are followed, the grain and resulting food produced will contain the required amounts of micronutrients to signpost to consumers.

### Seed standards and protocols

Seed standards are critical for identity preservation and aggregation in developing the supply chain for biofortified crops. *Certified seed* is defined by the USDA as “progeny of breeder, foundation or registered seed, handled under procedures acceptable to maintain satisfactory genetic purity and identity”;^72^ this definition has been adopted in all countries covered in our review. This is the critical stage in the value chain to ensure that the original seed comes from breeders and varieties that adhere to breeding targets; if the seeds are not genuinely biofortified varieties or have been mixed, then the entire value chain for the nutrient collapses and the value for value chain actors is lost:

Certification is the process by which a state seed certifying agency gives official recognition to seeds produced of a cultivar or named variety under a limited generation system which ensures genetic purity, identity, and a given minimum level of quality.^72^

There are different types of seed developed in the breeding process: breeders seed, foundation seed, registered seed, and certified seed. The latter class is commercial seed, and it produces grain that enters the food system, frequently through the private sector, initiating the food supply chain for NECs. All countries reviewed herein were found to have country‐specific seed standards or certified seed schemes. In addition, there are regional and global schemes, such as the Organization for Economic Co‐operation and Development (OECD) schemes for the Varietal Certification of Seed and Seed Codex^73^ in Nigeria that aims to increase crop productivity by tackling counterfeit seeds. The OECD is a good example of a process or set of standards to ensure the use of certified agriculture seed that is of consistently high quality.^74^

For certified seed, the genetic potential of the variety seed to express the micronutrient levels, as measured during the official registration and testing process, is guaranteed. The micronutrient content of the seed is not determined given the cost involved. When setting standards in the value chain, it is more important to set breeding standards and ensure that certified seed protocols are followed in identity preservation, and then test for nutrient content at grain level.

### Grain standards

**FIGURE 3:**
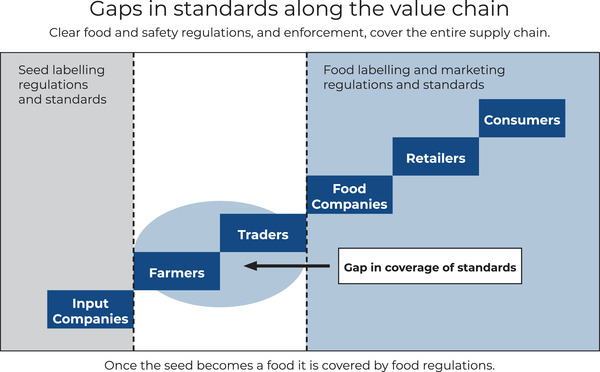
Gaps in standards along the value chain.

**FIGURE 4:**
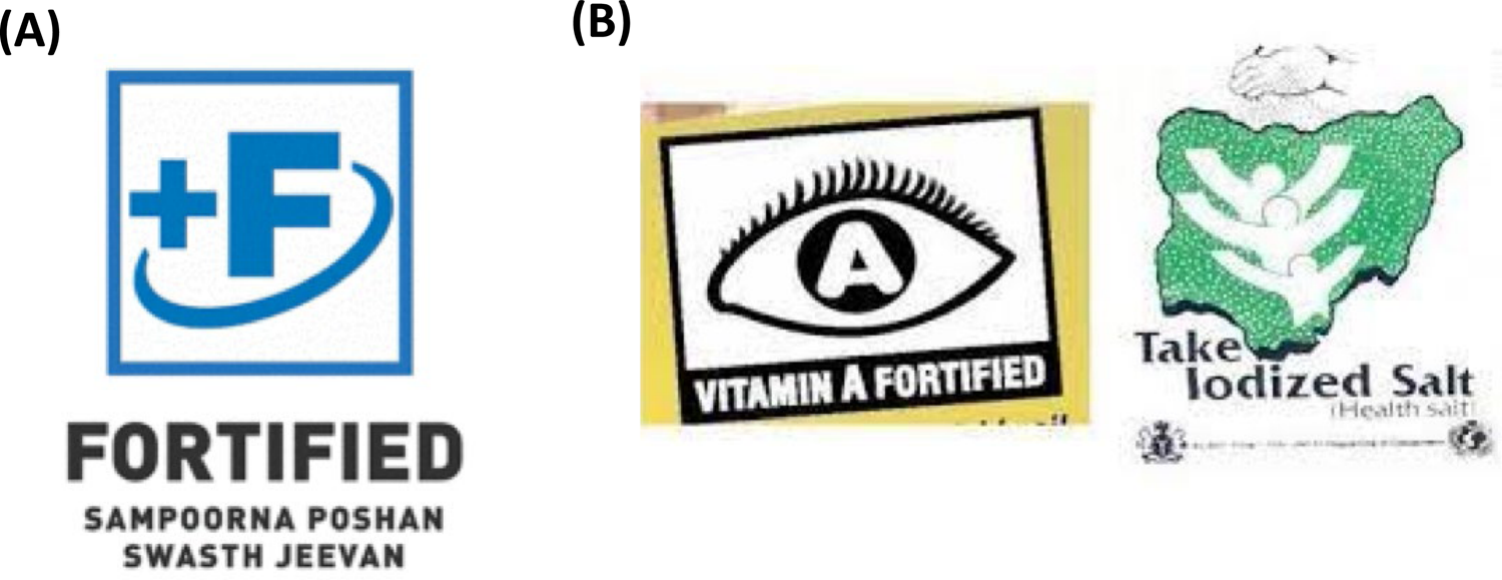
(A) Logo for fortified food under the Food Safety and Standards (Fortification of Foods) Regulations 2017. (B) Nigeria eye logo.

Regulations and standards play an important role in supporting commercialization. Across the various countries whose policies have been reviewed, there are clear gaps in regulatory standards for biofortified crops, particularly at the grain level (Figure 3, 4). There is significant regulatory oversight at the level of input traders. All priority countries have regulatory agencies that approve seeds or stems for sale within the country but not all of them have clear standards or certifications for biofortified seed. At the crop level involving farmers and traders, countries, in general, provide significantly less regulatory oversight. At this stage of the value chain, none of the priority countries have regulatory standards defining biofortified crops (i.e., a specific threshold of micronutrient per kg of crop) or a corresponding “certification” for biofortified crops. Additionally, no country currently oversees a certified testing program involving a system of certified laboratories licensed to test for micronutrient concentrations for biofortified crops. At the level of agroprocessors and retailers, prior to food reaching the consumers, there is moderate to significant regulatory oversight. Most countries have a local regulatory system of labeling standards for nutritional claims relating to processed agricultural products (e.g., source of a particular nutrient claim). In several countries (e.g., Pakistan and Bangladesh) that do not have specific regulations related to nutritional claims in processed foods, the global Codex Alimentarius rules still apply. While some countries (e.g., India) have integrated standards for biofortified products into their regulations for the labeling of fortified products, most countries are yet to reach that stage. This overall lack of common standards for biofortified crops impedes the development of segregated supply chains for these crops and creates significant sourcing and compliance uncertainty for downstream agriculture processors.

Stakeholder engagement conducted by HarvestPlus revealed that there is a significant need to work with regulators and standard experts to characterize, standardize, and establish a consistent set of rules to differentiate and communicate the benefits of biofortification from standard crops and ingredients. In general, standards are critical for major procurers of grain commodities—major buyers (private and institutional) cannot demand a product if it does not have a standard. Therefore, it became imperative to create an international, consensus‐driven system standard to define what makes *nutritionally enhanced* grains, roots, and tubers different from *standard* grains, roots, and tubers. Buyers would need to be specific with suppliers as to the type of grain they want to buy and then able to test if the grain meets the standards.

Various options for the creation of standards have been considered by HarvestPlus. The development of Codex standards does not have a cost to food business operators; however, working on them is resource‐intensive and time‐consuming. Moreover, Codex standards are rarely used for commercial procurement or in any buying standards, and Codex covers all technologies, including genetic engineering, and is open to industry pressures. An alternative to universal Codex standards would be the creation of individual standards for each crop and each country (e.g., iron enriched beans in Rwanda). A careful analysis of this approach revealed that this would require substantial resources and unnecessarily hinder trade by creating different national standards. The development of a unified global PAS through an International Standards Organization accredited body seems to be a rational step that could be efficiently adopted in all the priority country systems. However, the success of the PAS would depend on close partnership and support among country governments. Development and publication of each PAS specific for a given nutrient and covering all the crops enriched with that nutrient takes about a year. Since development of a PAS involves commercial leaders, the process ensures that the end users are aligned, and their inputs are taken as part of the process, this makes their development both cost‐ and time‐efficient and involves a fair and transparent process.

Some necessary considerations are important before proceeding with developing a PAS. Not all priority countries have a national standards body which could partner to develop a PAS, and those that do often lack sufficient expertise in nutrients of interest. Some of the evaluated country‐level standard bodies lacked efficient and transparent procedures and pricing policies. Creating standards in English was deemed an important consideration to facilitate easier translation into different languages.

The development of a set of PAS that cover the biofortified grains, across the nutrients that have been enriched for and applicable in the priority countries, has been initiated by HarvestPlus. The standards, *PAS 233: Zinc enriched wheat, maize, and rice grain – Specification* ^68^ and *PAS 234: Iron enriched bean and pearl millet grain – Specification*,^69^ provide important tools for private and public enterprises in the food supply chain that procure and sell grain for human consumption, as well as governments, and associated quality and compliance agencies. Work is already underway on a third PAS—for vitamin A maize, cassava, and sweet potato—with publication expected in July 2022. These standards will set out good practice requirements for class levels of zinc and iron concentration, sampling guidance, packaging, and labeling. By specifying the parameters of increased nutrient content in seeds obtained by conventional farming methods, these standards are intended to enable greater transparency in the food value chain. Governments and their agencies wishing to improve nutrition in staple crops and resulting foods for their population's health and wellbeing will be able to follow the good practice set out in the standards, and organizations producing food products using these crops can be assured of their provenance.

### Standards and regulations for foods‐ practical applications for food packaging

Once breeding standards have been followed, certified seed protocols adhered to, and certified seed demanded by farmers and the crop has been grown and tested at grain level, the final step in the chain is the production of food. If all previous steps have been followed correctly, the nutrient will be present in the food. Previous reviews had not found food labeling regulations for biofortification. In 2017, Garcia‐Casal et al.^75^ concluded that “Regulatory considerations are rare or nonexistent for biofortification by conventional plant breeding.” In the same year, Mejia et al.^6^ found that “the production and marketing of these [biofortified] products [foods] have been conducted without regulatory framework and under limited government control or regulatory guidance.” Following the Codex deliberations on the definition and subsequent affirmation that existing food labeling provisions, codex texts, standards, and regulations already covered biofortified grains and foods and their products thereof, we found that in all countries reviewed adequate food labeling provisions exist to promote NEC and foods made from NEC (Table 1).

Nutrient content claims are simple messages to show a consumer that a food contains meaningful quantities of a nutrient, for example, claims, such as “source of iron” can be made when a food contains approximately 15% of the NRV. Most countries in Europe, Africa, and Asia permit claims based on 100 g of food, whereas countries in North America, Canada, and Latin America follow “a per portion” system or reference amount customarily consumed; if the food is eligible to make a nutrition claim, then most countries’ legislation permit a health claim—a health claim refers to the role or function of the nutrient, such as “iron contributes to normal cognitive development of children.” Such nutrition claims should be based on the textbook or generally accepted function of the nutrient. The most comprehensive list or register of nutrient function claims is found in “Regulation (EC) No 1925/2006 of the European Parliament and of the Council of 20 December 2006 on the addition of vitamins and minerals and of certain other substances to foods.”^62^

While biofortification was intended for malnourished communities that rely on large quantities of a single food source, nutrient levels of most biofortified crops permit nutrition and health claims in finished foods. This depends on the final food. Producers of the simple food format of the staple, such as flour, bread, or brown rice, are in most cases able to make claims to consumers; however, once the biofortified grain is “diluted” by other ingredients in composite foods, the nutrient may not be in sufficient quantities to make nutrition and health claims. The nutrient content of the food can initially be calculated by using nutritional databases, such as McCance and Widdowson,^76^ or the USDA Food Data Central.^77^ However, when making this calculation, the nutrient values of the biofortified crop will need to be entered manually using data obtained from the nutrient‐specific PAS or from HarvestPlus; the values used in standard data sets are not from biofortified varieties. Table 2 provides approximate values of the nutrient per biofortified crop. It is advisable that finished food products are also analyzed in a laboratory using accredited methods to ascertain the final amount of the micronutrient. Food should be tested on a regular basis throughout the shelf life of the food and crop on a seasonal basis. These costs are often too high for small and medium‐sized enterprises (SMEs) in low‐ and middle‐income countries (LMICs).

**TABLE 2:** Approximate nutrient values of selected biofortified foods

Nutrient	Crop	Unit	Level per 100 g	Labeling RNIs	Notes
Zinc	Wheat	mg	3.5	10	Whole zinc wheat India average
Zinc	Rice	mg	1.7	10	Brown zinc rice India average
Zinc	Maize	mg	3.0	10	Wholegrain zinc maize Colombia
Iron	Beans	mg	9.5	14	Black ICTA Chorti‐ACM Iron bean
Iron	Pearl millet	mg	7.4	14	Wholegrain Iron pearl India
Vitamin A	Sweet potato	μg	700	800	Use PAS target value
Vitamin A	Maize	μg	137	800	Zambia. Wholegrain and wet values
Vitamin A	Casava	μg	160	800	Nigeria, IITA‐TMS – IBA154810

*Note*: Values in table above are for illustration and from specific varieties in countries. Producers looking for specific values should consult their supplier or HarvestPlus for more detailed information.

There is a clear pathway to ascertain if nutrition or health claims can be made under all countries evaluated in this review. Knowing what the nutritional function is then provides a route to name the product. Since the word biofortification was not found in any regulatory documents and is also unknown to most consumers, it is highly recommended not to use the word biofortification in any consumer facing materials, including the packaging. The product should be clearly named using the name of the nutrient, for example, “vitamin A maize, flour” or “vitamin A maize, corn flakes.”

It is possible to communicate to the consumer that the food is a natural source of the vitamin. However, it is important not to imply that the product itself is natural or contains natural ingredients, especially if it is a composite food. Natural foods and natural food ingredients are often subject to separate rules and guidelines, such as those found in the UK^78^ or the United States. In the absence of any country‐specific rules, these can be followed as a comprehensive approach to protect the consumer.

Logo or signpost schemes also exist in several markets, in India provisions to use the F+ logo are available as part of the promotion of fortified foods to consumers; this logo could be used on biofortified wheat and rice products^79^ and in Nigeria, it is possible to use the eye logo for vitamin A products (Figure 4).^80^ HarvestPlus is also in the process of creating and registering a consumer logo.

It is not always possible to make nutrition and health claims and then subsequently, signpost the nutritional benefits to the consumer. There are other ways in which populations benefit from the use of NEC foods in food systems. The use of biofortified grains/roots in processed foods creates demand. This demand then stimulates farmers of all sizes to grow biofortified foods, for smallholders, this increases the availability of nutritious foods to be consumed on farm and simultaneously ensures more nutritious foods in local food systems. In many instances, especially in this rapid‐scale phase of biofortification, consumers will be consuming biofortified commodities without knowing it. Known as the *fluoride in the water* approach, consumers will be buying and eating their regular commodities, which have the added benefit of more nutrition. For the vitamin A crops which are visually different, more consumer awareness campaigns might be required. When it is not possible to make nutrition or health claims, food processors will require other incentives to use biofortified commodities, this could be in the form of corporate social responsibility programs, environmental, social, and corporate governance commitments, or impact investing. It is also possible to communicate to the consumer the benefits of partnering with the biofortification movement without making implied nutritional claims. HarvestPlus has produced guidelines to manufacturers on ways to promote biofortified products.^18^ Guidelines are being produced for India, Nigeria, Pakistan, and Zimbabwe and expected to be disseminated to manufacturers in 2022.

Biofortification was designed to prevent and treat malnutrition, but when it comes to the promotion of foods to consumers, it is paramount not to make claims about disease prevention or cure. The benefits of the nutrients should be positive, such as building and maintaining health. Some countries, such as the EU, do permit disease risk reduction claims, but they are generally approved on a case‐by‐case basis with a lengthy approval process and dossier of population‐specific evidence.

Nutrition and health benefits of biofortified foods should not be made on processed foods, which are high in fat, salt, or sugar. While the debate continues as to what makes a food unhealthy, foods of this nature should not be overly promoted. These foods should still be made using biofortified commodities but not promoted by the benefits. Limiting biofortified foods to only healthy foods would create divides in supply chains and negatively affect consumers with the poorest diets.

### Enforcement of standards and regulations

Enforcement is rare and often limited to high‐income countries and mass marketed foods. Trading standards, advertising standards, and consumer protection are often unavailable, and breaches of rules are highlighted on high‐volume foods that are heavily promoted and, therefore, attract attention. Little attention is paid to the way that SMEs promote their products in LMICs, therefore, it is essential to provide regulatory advice and support to growing businesses aiming to develop healthy affordable foods. Partners in Food Solutions is an independent, nonprofit organization, which supports business in Africa with services, such as labeling. HarvestPlus has also released white papers to support businesses.

All food businesses should be advised on consumer protection and making nutrition and health claims that promote the products but are completely truthful and helpful to the consumer. Sticking to the facts about the food is always safe advice to provide food marketers.

### Consumer understanding and best methods to promote to consumers

Foti et al.^81^ concluded that consumers of biofortified food products are generally confused and uninformed, even though they show a high willingness to pay. This confusion seems to result, moreover, from the lack of a clear definition of a biofortified product. The same conclusion was found by Timpanaro et al.,^82^ who concluded that “at present the potential consumer of biofortified food products is generally confused and uninformed.” These findings were due to the use of the term biofortification and would not have found the same conclusions had the term biofortification been removed. Research conducted by Leatherhead food international in 2018^16^, ^83^ found that 88% of consumers rated the presence of naturally occurring vitamins and minerals to be fairly or very important. Naturally nutritious, naturally healthy packaged foods continue to grow year on year. Given the global trend for natural foods, clean labels, and interest in protecting immunity, biofortified foods are in prime position to grow.^84^ Consumers in LMICs consistently show positive outcomes for biofortified foods, even for the orange crops with visible differences. In sensory evaluations and willingness to pay, studies show that consumers accept the food and in many cases are willing to pay more. Studies in rural Uganda revealed that when nutrition information on the benefits of vitamin A sweet potato was provided, consumers valued the vitamin A‐rich orange varieties more than white ones.^85^, ^86^ More recently, Rizwan et al.^87^ found that there is significant scope for promoting zinc wheat in the country.

Positioning of biofortified foods is fundamental to consumer acceptance. Promote the benefits of NECs and not the biofortification process, focus on natural nutrition, clean label, and the ethical story. Consumer research assessing current consumer understanding of biofortification becomes irrelevant when the term biofortification is removed. Manufacturers should be advised not to use the term biofortification: it is misleading to consumers and could also potentially breach local or regional food labeling regulations by using terminology unknown to consumers or lacking any regulatory endorsement; but most importantly, the term will not be used to promote or sell the product to the consumer.

## CONCLUSIONS

We have found that in every country evaluated, standards exist at each step of the value chain to enable trade and marketing of nutrient enriched seeds, grains, and foods, especially with the publication of the PAS for grain. Standards, labeling, and marketing for biofortified foods are not complicated as once thought. Even with the long value chain (from agricultural research to processed foods) and the many consumption points from farming family consumption, sale of the basic commodity (flour), minimally processed foods, such as bread, and more highly processed, composite foods. NECs are commonly consumed staples and infiltrate the food system in most processed food categories. Previous thinking on searching for regulations for biofortified foods was hampered using the term biofortification. In all this research, the conclusions of Mejia et al.^6^ still stand, namely, that foods from conventional bred foods do not require any special regulatory provisions. All other technologies, such as genetic engineering, are governed by specific regulations. Biofortification has moved on rapidly since the inception of the HarvestPlus program in 2004; consequently, the term biofortification should not be used for regulatory research or food products, for assessing consumer demand, or for food product labeling. The name of the nutrient and the name of the crop of interest should be used for search terms and consumer testing. The term biofortification should only be used to describe the process for academic or policy purposes. Research that concluded that the consumers are confused by the term biofortification—while correct (i.e., consumers do not like the word)—should not be used to guide food manufacturers or policy makers in this area. Our view here concludes that standards and regulations are available at every step of the value chain to allow and promote trade of commodities from NECs using conventional breeding, and there is a growing consumer need and demand for naturally nutritious foods. NECs are ripe for growth across the world.

## AUTHOR CONTRIBUTIONS

T.M.‐G.: cowriting the regulatory framework of the manuscript, lead researcher, subject matter expert, and lead writing on PAS section and writing in all other sections, editing, and revising. W.H.P.: subject matter expert in agronomy, breeding, and seed systems, editing, contribution to conceptualization, contribution to summary, conclusions, and implications. J.W.: conceptualization of the research and outline of the paper, writing the framework of the paper, lead researcher on methodology, regulatory review, and lead writing on several sections.

## COMPETING INTERESTS

Wolfgang H. Pfeifer and Jenny Walton work for the HarvestPlus team in the CGIAR. The principal donors for the HarvestPlus program are the UK Government, the Bill & Melinda Gates Foundation, the Government of Canada, the U.S. Government's Feed the Future Initiative, the Children's Investment Fund Foundation, and the John D. and Catherine T. MacArthur Foundation.

## References

[1] Biofortification of staple crops . https://www.who.int/elena/titles/biofortification/en/

[2] Golden Rice Project . https://www.goldenrice.org/

[3] The Mushroom Council . https://www.mushroomcouncil.com/vitamin‐d/

[4] Barnett, A. (2015). The nature of crops: Why do we eat so few of the edible plants? NewScientist, Accessed on Dec 2021, https://www.newscientist.com/article/mg22730301-400-the-nature-of-crops-why-do-we-eat-so-few-of-the-edible-plants/

[5] 2021 Global Nutrition Report . https://globalnutritionreport.org

[6] Mejia, L. A. , Dary, O. , & Boukerdenna, H. (2017). Global regulatory framework for production and marketing of crops biofortified with vitamins and minerals. Annals of the New York Academy of Sciences, 1390, 47–58. 27801985 10.1111/nyas.13275

[7] HarvestPlus. (2018). Our history. www.harvestplus.org/about/our‐history

[8] Talsma, E. F. , & Pachón, H. (2017). Biofortification of crops with minerals and vitamins. https://www.who.int/elena/titles/bbc/biofortification/en/#:~:text=Biofortification%20is%20the%20process%20by,importantly%20to%20farmers%20

[9] USDA NAL Agricultural Thesaurus and glossary . https://agclass.nal.usda.gov/mtwdk.exe?s=1&n=1&y=0&l=60&k=glossary&t=2&w=biofortification

[10] Codex Alimentarius . Report of the 39th Session of the Committee on Nutrition and Foods for Special Dietary Uses, 4–8 December 2017. http://www.jhnfa.org/k173.pdf

[11] Report of the 40th Session of the Committee on Nutrition and Foods for Special Dietary Uses, 26–30 November 2018 . http://www.fao.org/fao‐who‐codexalimentarius/sh‐proxy/en/?lnk=1&url=https%253A%252F%252Fworkspace.fao.org%252Fsites%252Fcodex%252FMeetings%252FCX‐720‐40%252FREPORT%252FREP19_NFSDUe.pdf

[12] Responding to Crisis, Building Resilience: The 2020 HarvestPlus Annual Report . https://www.harvestplus.org/knowledge‐market/in‐the‐news/responding‐crisis‐building‐resilience‐2020‐harvestplus‐annual‐report

[13] Improving nutrition through biofortification: From strategy to implementation . https://www.cerealsgrains.org/publications/cfw/2019/May‐June/Pages/CFW‐64‐3‐0026.aspx

[14] Bouis, H. E. , & Welch, R. M. (2010). Biofortification—A sustainable agricultural strategy for reducing micronutrient malnutrition in the Global South. The future of food: Developing more nutritious diets and safer food. 10.2135/cropsci2009.09.0531 https://acsess.onlinelibrary.wiley.com/doi/10.2135/cropsci2009.09.0531

[15] Titoria, P. , Gubisch, E. , Leedam, O. , & Linsley, S. (2018). Mainstreaming biofortified crops. https://www.harvestplus.org/sites/default/files/leatherheadfinal%20report.pdf

[16] Differentiating and communicating biofortified products in the current regulatory landscape . https://www.harvestplus.org/sites/default/files/Differentiating%20and%20Communicating.pdf

[17] 2FAS: About us . https://www.2fas.org/

[18] Nutrient‐enriched crops . https://www.2fas.org/_files/ugd/613086_e5736d655bfb4c09ab4706ca4f0a322a.pdf

[19] Codex Alimentarius International Food Standards . Biotechnology. https://www.fao.org/fao‐who‐codexalimentarius/thematic‐areas/biotechnology/en/

[20] GMO Legislation. https://ec.europa.eu/food/plants/genetically‐modified‐organisms/gmo‐legislation_en

[21] U.S. Food & Drug Administration . Agricultural biotechnology. https://www.fda.gov/food/consumers/agricultural‐biotechnology

[22] Plan National d'Investissement Agricole (PNIA) 2014 . http://extwprlegs1.fao.org/docs/pdf/cng146463.pdf

[23] Kenya National Nutrition Action Plan 2018–22 . https://scalingupnutrition.org/wp‐content/uploads/2020/10/Kenya‐National‐Nutrition‐Action‐Plan‐2018‐22.pdf

[24] Global database on the Implementation of Nutrition Action (GINA) . https://extranet.who.int/nutrition/gina/es/node/57262

[25] Kenya Standards KS EAS 805:2014 . https://webstore.kebs.org/index.php?route=product/product&product_id=10946

[26] Food fortification in Kenya Policy Brief . https://www.nutritionintl.org/wp‐content/uploads/2020/12/Kenya‐Food‐Fortification_FINAL_2020‐11‐02_WEB‐1.pdf

[27] Policy ‐ National Plan of Action on Food and Nutrition in Nigeria . https://extranet.who.int/nutrition/gina/en/node/7943

[28] National Agency for Food and Drug Administration and Control (NAFDAC) . Pre‐packaged Food, Water and Ice Labelling Regulations, 2019. https://www.nafdac.gov.ng/wp‐content/uploads/Files/Resources/Regulations/New_Draft_Regulations/Pre‐Packaged‐Food‐Water‐and‐Ice‐Labelling‐Regulations‐2019.pdf

[29] Food fortification in Kenya Policy brief . https://www.nutritionintl.org/wp‐content/uploads/2020/12/Kenya‐Food‐Fortification_FINAL_2020‐11‐02_WEB‐1.pdf

[30] Tanzania National Biofortification guidelines . https://www.kilimo.go.tz/uploads/dasip/English_version_Final_new2.pdf

[31] (2011). Policy ‐ The Tanzania Food, Drugs and Cosmetics (Food Fortification) Regulations.

[32] Global database on the Implementation of Nutrition Action (GINA) . https://extranet.who.int/nutrition/gina/en/node/14767

[33] Biofortification in the Uganda Nutrition Action Plan (II) under review . Accessed on July 2022, https://opm.go.ug/second-uganda-nutrition-action-plan-passed/

[34] Uganda Nutrition Action Plan (UNAP) . https://www.health.go.ug/docs/UNAP_11_16.pdf

[35] The Uganda National Bureau of Standards Act (Declaration of Compulsory National Standards) Notice . (2015). https://extranet.who.int/nutrition/gina/en/node/57264

[36] The Food and Drugs (Food Fortification) (Amendment) Regulations . (2011) http://ugandanlawyer.com/wp‐content/uploads/2019/03/Food‐and‐drugs‐food‐fortification‐regulations‐2011.pdf

[37] Rwanda Standard RS 350. https://members.wto.org/crnattachments/2017/TBT/RWA/17_3857_00_e.pdf

[38] International Trade Administration Labeling and Marking requirements. https://www.trade.gov/country‐commercial‐guides/rwanda‐labeling‐and‐marking‐requirements

[39] Policy ‐ Standards of the Rwanda Standards Board (RSB): National Standards. https://extranet.who.int/nutrition/gina/en/node/57265

[40] Food fortification regulation in Rwanda. https://static1.squarespace.com/static/5e1df234eef02705f5446453/t/5f3d6ab1c2dd925afe1dd604/1597860530027/Day2‐3‐3Rwanda+.pdf

[41] National Food and Nutrition Strategic Plan‐2017 to 2021. https://www.nfnc.org.zm/download/national‐food‐and‐nutrition‐strategic‐plan‐2017‐to‐2021/

[42] Government of Zambia Food and Drugs Act. https://extranet.who.int/nutrition/gina/sites/default/filesstore/ZMB%201972%20Food%20and%20Drugs%20Act.pdf

[43] The National Food and Nutrition Commission (NFNC) Zambia. https://www.nfnc.org.zm/

[44] Republic of Zambia Food and Drugs Act. https://www.parliament.gov.zm/sites/default/files/documents/acts/Food%20and%20Drugs%20Act.pdf

[45] Zimbabwe National Food Fortification Strategy . (2022 – 2026) Draft. Zimbabwe Ministry of Health and Child Welfare. FAO and UNICEF (in preparation). Accessed on July 2022, https://executiveboard.wfp.org/document_download/WFP-0000137413

[46] Zimbabwe the Food and Food Standards Act 15:04 and Regulations of 2002 . https://www.ecolex.org/details/legislation/food‐and‐food‐standards‐act‐chapter‐1504‐lex‐faoc024975/#:~:text=An%20Act%20to%20to%20provide,food%20and%20matters%20incidental%20thereto

[47] Zimbabwe Food Fortification Regulations . (2016). http://www.veritaszim.net/sites/veritas_d/files/SI%202016‐120%20‐%20Food%20Fortification%20Regulations%2C%202016.pdf

[48] Compact 2025 Bangladesh ending hunger & undernutrition. Challenges & opportunities. https://www.compact2025.org/files/2016/04/Bangladesh‐Scoping‐Report_Final.pdf

[49] Bangladesh Packaged Food Labeling Act 2017. https://www.fas.usda.gov/data/bangladesh‐bangladesh‐issues‐packaged‐food‐labelling‐act‐2017

[50] Bangladesh Standard for guidelines for use of nutrition and health claims. https://extranet.who.int/nutrition/gina/sites/default/filesstore/BGD%202008%20Nutrition%20and%20Health%20Claims.pdf

[51] Mullen, A. (2021). Industry compliance to fortification in Bangladesh. Nature Food, 2, 637. 10.1038/s43016-021-00378-7 37117474

[52] Indian Council of Agricultural Research . New Delhi biofortified varieties: Sustainable way to alleviate malnutrition. https://icar.org.in/files/BiofortifiedEnglish_.pdf

[53] Food Safety and Standards (Packaging and Labelling) Regulations . (2011). https://www.fssai.gov.in/upload/uploadfiles/files/Packaging_Labelling_Regulations.pdf

[54] Food Safety and Standards (Advertising and Claims) Regulations . (2018). https://fssai.gov.in/upload/uploadfiles/files/Compendium_Advertising_Claims_Regulations_04_03_2021.pdf

[55] Food Safety and Standards (Fortification of Foods) Regulations . (2018). https://www.fssai.gov.in/upload/uploadfiles/files/Compendium_Food_Fortification_Regulations_30_09_2021.pdf

[56] Pakistan, National IRMNCAH&N Strategy (2016–2020). https://phkh.nhsrc.pk/sites/default/files/2019‐06/National%20RMNCAH&N%20Strategy%202016‐2020.pdf

[57] The West Pakistan Pure Food Ordinance . (1960). https://phkh.nhsrc.pk/sites/default/files/2021‐01/Pure%20Food%20Ordinance%20West%20Pakistan%201960.pdf

[58] Punjab Pure Food Regulations . (2018). https://foodscienceuniverse.com/wp‐content/uploads/2020/10/Punjab‐Pure‐Food‐Regulations‐2018.pdf

[59] Food Fortification Advisory Service (2FAS). (2020). Nutrient enriched crops. https://www.2fas.org/_files/ugd/613086_e5736d655bfb4c09ab4706ca4f0a322a.pdf

[60] Regulation (EU) No 1169/2011 of the European Parliament and of the Council of 25 October 2011 on the provision of food information to consumers. https://eur‐lex.europa.eu/legal‐content/EN/ALL/?uri=CELEX%3A32011R1169

[61] Regulation (EC) No 1924/2006 of the European Parliament and of the Council of 20 December 2006 on nutrition and health claims made on foods. https://eur‐lex.europa.eu/legal‐content/EN/TXT/?uri=CELEX:02006R1924‐20141213

[62] Regulation (EC) No 1925/2006 of the European Parliament and of the Council of 20 December 2006 on the addition of vitamins and minerals and of certain other substances to foods. https://eur‐lex.europa.eu/legal‐content/EN/ALL/?uri=CELEX:32006R1925

[63] Lockyer, S. , White, A. , Walton, J. , & Buttriss, J. L. (2018). Proceedings of the ‘Working Together to Consider the Role of Biofortification in the Global Food Chain’ workshop.

[64] Food labelling and packaging. https://www.gov.uk/food‐labelling‐and‐packaging

[65] Fortified foods: Adding vitamins, minerals and certain other substances. https://www.gov.uk/government/publications/fortified‐foods‐guidance‐to‐compliance‐with‐european‐regulation‐ec‐no‐1925‐2006‐on‐the‐addition‐of‐vitamins‐and‐minerals‐and‐certain‐other‐substances‐to‐food

[66] Estrategia Nacional Para La Prevención Y Control De Las Deficiencias De Micronutrientes En Colombia 2014 – 2021. https://www.minsalud.gov.co/sites/rid/Lists/BibliotecaDigital/RIDE/VS/PP/SNA/Estrategia‐nacional‐prevencion‐control‐deficiencia‐micronutrientes.pdf

[67] Resolución 333/2011. https://fedepanela.org.co/gremio/descargas/resolucion‐333‐de‐2011/

[68] *PAS 233: Zinc enriched wheat, maize and rice grain – Specification*. https://shop.bsigroup.com/products/zinc‐enriched‐wheat‐maize‐and‐rice‐grain‐specification/standard

[69] *PAS 234: Iron enriched bean and pearl millet grain – Specification*. https://shop.bsigroup.com/products/iron‐enriched‐bean‐and‐pearl‐millet‐grain‐specification/standard

[70] Virk, P. S. , Andersson, M. S. , Arcos, J. , Govindaraj, M. , & Pfeiffer, W. H. (2021). Transition from targeted breeding to mainstreaming of biofortification traits in crop improvement programs. Frontiers in Plant Science, 12, 703990. 10.3389/fpls.2021.703990 34594348 PMC8477801

[71] Biofortified varieties: Sustainable way to alleviate malnutrition. https://icar.org.in/files/BiofortifiedEnglish_.pdf

[72] USDA. (2009). Understanding seed certification and seed labels. https://www.nrcs.usda.gov/Internet/FSE_PLANTMATERIALS/publications/lapmctn9030.pdf

[73] SEEDCODEX is boosting crop productivity by tackling counterfeit seeds in Nigeria. https://agra.org/ourharvest/july‐20/seedcodex‐is‐boosting‐crop‐productivity‐by‐tackling‐counterfeit‐seeds‐in‐nigeria/

[74] The Organisation for Economic Co‐operation and Development (OECD). Promoting the use of certified agriculture seed. OECD Seed Scheme Rules, Regulations and Guidelines. https://www.oecd.org/agriculture/seeds/rules‐regulations/

[75] Garcia‐Casal, M. , Pena‐Rosas, J. P , Giyose, B. , Melse‐Boonstra, A. , & Talsma, E. (2017). Staple crops biofortified with increased vitamins and minerals: Considerations for a public health strategy. Annals of the New York Academy of Sciences, 1390, 3–13. 27936288 10.1111/nyas.13293

[76] Composition of foods integrated dataset (CoFID) McCance and Widdowson's ‘composition of foods integrated dataset’ on the nutrient content of the UK food supply. https://www.gov.uk/government/publications/composition‐of‐foods‐integrated‐dataset‐cofid

[77] USDA Food Data Central. https://fdc.nal.usda.gov/

[78] Use of the term natural on food labeling. https://www.fda.gov/food/food‐labeling‐nutrition/use‐term‐natural‐food‐labeling 10.1111/1750-3841.15128 32449960

[79] Food Safety and Standards (Fortification of Foods) Regulations . (2018) https://www.fssai.gov.in/upload/uploadfiles/files/Compendium_Food_Fortification_Regulations_30_09_2021.pdf

[80] Food Fortifications Regulations, 2021 ‐ NAFDAC . https://www.nafdac.gov.ng/wp‐content/uploads/Files/Resources/Regulations/REGULATIONS_2021/FOOD‐FORTIFICATION‐REGULATIONS‐2021.pdf

[81] Foti, V. T. , Scuderi, A. , Bellia, C. , & Timpanaro, G. (2021). Biofortification of tomatoes in Italy: Status and level of knowledge. Agricultural Economics – Czech, 67, 227–235.

[82] Timpanaro, G. , Bellia, C. , Foti, V. T. , & Scuderi, A. (2020). Consumer behaviour of purchasing biofortified food products. Sustainability, 12(16), 6297.

[83] Walton, J. (2019). Working with the food industry to expand biofortification's reach. https://www.harvestplus.org/knowledge‐market/in‐the‐news/working‐food‐industry‐expand‐biofortifications‐reach

[84] Euromonitor . (2022). Naturally healthy packaged food in the US. https://www.euromonitor.com/naturally‐healthy‐packaged‐food‐in‐the‐us/report

[85] Birol, E. , Meenakshi, J. V. , Oparinde, A. , Perez, S. , & Tomlins, K. (2015). Developing country consumers’ acceptance of biofortified foods: A synthesis. Food Security, 7(3), 555–568.

[86] Chowdhury, S. , Meenakshi, J. V. , Tomlins, K. I. , & Owori, C. (2011). Are consumers in developing countries willing to pay more for micronutrient‐dense biofortified foods? Evidence from a field experiment in Uganda. American Journal of Agricultural Economics, 93(1), 83–97.

[87] Rizwan, M. , Zhu, Y. , Qing, P. , Zhang, D. , Ahmed, U. I. , Xu, H. , Iqbal, M. A. , Saboor, A. , Malik, A. M. , Nazir, A. , Wu, X. , He, P. , & Tariq, A. (2021). Factors determining consumer acceptance of biofortified food: Case of zinc‐fortified wheat in Pakistan's Punjab Province. Frontiers in Nutrition, 8, 647823. 34179055 10.3389/fnut.2021.647823 PMC8220091

